# Long COVID and mental health correlates: a new chronic condition fits existing patterns

**DOI:** 10.1080/21642850.2022.2164498

**Published:** 2023-01-08

**Authors:** Michael L. Goodman, Stephen Molldrem, Aleisha Elliott, David Robertson, Philip Keiser

**Affiliations:** a Deparment of Internal Medicine, University of Texas Medical Branch, USA; b Department of Preventive Medicine and Population Health, University of Texas Medical Branch, USA; c Area Health Education Center, University of Texas Medical Branch, USA; d Yes& Lab, Houston, TX, USA; e Galveston County Health District, USA

**Keywords:** Long COVID, chronic diseases, Common mental disorders, suicide ideation, PTSD

## Abstract

**Background:**

Emerging Long COVID research indicates the condition has major population health consequence. Other chronic conditions have previously been associated with functional and mental health challenges – including depression, anxiety, post-traumatic stress disorder (PTSD), suicide ideation, substance use and lower life satisfaction.

**Methods:**

This study explores correlations between self-reported Long COVID, functional and mental health challenges among a random community-based sample of people (*n* = 655) aged 20–50 years who contracted COVID-19 prior to vaccination in a Texas county. A random sample of eligible participants was mailed a link to participate in a semi-structured questionnaire. Participant responses, including open-ended responses regarding their experience following COVID-19, were paired with health system data.

**Results:**

Long COVID was associated with increased presence of depression (13% increase), anxiety (28% increase), suicide ideation (10% increase), PTSD (20% increase), and decreased life satisfaction and daily functioning. Structural equation modeling, controlling for sociodemographic variables and imposing a theoretical framework from existing chronic disease research, demonstrated correlations between Long COVID and higher PTSD, suicide ideation and lower life satisfaction were mediated by higher daily functional challenges and common mental disorders.

**Conclusions:**

Basic and applied, interdisciplinary research is urgently needed to characterize the population-based response to the new challenge of Long COVID.

## Background

Mental health challenges often co-occur with chronic physical health conditions (e.g. Manning et al., 2019). As researchers increasing focus on Long COVID, it is important that we understand the complete picture of the mental health of Long COVID patients alongside physiological etiologies.

Long COVID^1^ has been identified across patients of all ages, and geographic regions (Bossley et al., 2022; Kuodi et al., 2022; Li et al., 2020; Mehandru & Merad, 2022; Mendelson et al., 2021; Penetra et al., 2022; Say et al., 2021; Tohamy et al., 2021; Vanichkachorn et al., 2021). The US Centers for Disease Control and Prevention (CDC) estimates that 19% of all American adults who previously tested positive for COVID-19, or 7.5% of all Americans, currently report Long COVID symptoms (National Center for Health Statistics, 2022; NCHS, 2022). While definitional issues persist surrounding Long COVID, self-reported and clinical evidence of Long COVID indicate the need for ongoing studies about what and how this novel condition manifests (Mol, 2002).

Long COVID may be associated with worse mental health (Aiyegbusi et al., 2021). Other chronic conditions, such as chronic fatigue syndrome, chronic pain, physical disability, and HIV, are associated with worse mental health (Gatchel, 2004; Manning et al., 2019; Nater et al., 2009; Remien et al., 2019; Tough et al., 2017). Disruptions to daily functioning explain some of the associations between chronic physical condition and worsened mental health outcome – including depression, and anxiety (Dean et al., 2010; Gullo et al., 2019; Remien et al., 2019). Higher depression mediates associations between chronic conditions and post-traumatic stress disorder (PTSD; Morasco et al., 2013; Outcalt et al., 2015).

The associations between worse mental health, physical health and daily functioning appear to be antagonistic and recursive, potentially contributing to a downward spiral implicating mental, behavioral, and physical well-being (Okifuji & Turk, 2016). Downward spirals involving multiple life domains can predict suicide ideation and behavior, as individuals experience what they perceive to be a hopeless spiral of despair in a life to which they no longer feel they belong (Shim et al., 2019; Wilson et al., 2013; 2017). Higher substance abuse, and lower life satisfaction, are common in such downward spirals (Centanni et al., 2019; Garland et al., 2013; Oishi et al., 2009).

Thus, while research related to Long COVID is very young, the ubiquity, heterogeneity, and novelty of Long COVID call for additional research to understand whether this novel condition parallels other known chronic conditions with respect to mental health. Ongoing activism surrounding Long COVID and calls for its recognition also stand in a long tradition of patient self-advocacy, from which scientific investigations can take cues (Callard & Perego, 2021; Epstein, 1995).

### Study aim

This study aims to identify associations between self-reported Long COVID and worse mental health within a population-based sample in Galveston County, Texas through a survey administered during April and May 2022.

Structural Equation Models were used to assess the extent to which observed correlations may be explained by the following hypothesized pathway: Long COVID symptom, worse daily functioning, common mental disorder (anxiety & depression), PTSD, suicide ideation, alcohol use and life satisfaction.

Qualitative responses from an open-ended question at the end of the survey were analyzed using a deductive coding approach within a thematic analysis framework (Braun & Clarke, 2006). The open-ended response at the end of the survey asked: ‘What else would you like people to know about your experience of COVID-19, including, possibly, your experience of long COVID symptoms?’ The quantitative findings were used to identify quotations of interest and to create a list of deductive codes that the second author used to explore the qualitative end-of-survey responses and to select demonstrative quotations. We sought to identify substantive quotes that enhanced the meaning of the quantitative findings.

## Methods

### Study setting

Galveston County is in southeast Texas, has a population around 350,000 and is home to University of Texas Medical Branch. Recently strengthened governmental digital health data management system permitted linking variables within the present dataset.

### Sample selection

The sample for this study was selected using a multi-stage sampling approach to account for (1) expected gender differences in Long COVID, (2) likely differences in Long COVID risk by vaccine status, (3) potential geographic variation within Galveston County and (4) particular concern for families and potential for intergenerational risks of sequelae to COVID-19 disease. First, beginning with the public health surveillance data of all persons who were reported by official lab report to Galveston County Health District as having tested positive for SARS-CoV-2, we excluded all people who tested positive for COVID-19 after receiving any vaccine dose. Second, we excluded anyone who was outside the age range of 20–50 years, leaving an initial potential sample of 49,542 individuals. We limited our sample to this age range to increase the probability of a child living in the respondent’s household due to a concern for potential intergenerational sequelae of Long COVID.^2^ Third, we determined a sample size of 6,250 women and 3,135 men to account for an anticipated gender difference in (1) probability of Long COVID and (2) child rearing duties. The total sample size of 9,500 was selected due to an expected response rate near 7% and funding limits (Fox et al., 1988). The sample size was stratified proportionate to the contribution of each municipality to the total prevalence of individuals between the ages of 20–50 who tested positive for COVID-19 prior to receiving a vaccine within Galveston County.

The sample size was not predetermined, given the status of the study as an exploratory effort to assess correlates of Long COVID. The sample size was limited by budgetary constraints, but adequate for power calculations presented here.

### Procedure

Each selected person was mailed an invitation to participate in the surveillance study, a unique ID number, a commitment to receive $40 upon completion of the study, and a hyperlink (QR code and URL link) to a survey instrument with standardized measures hosted on REDCap (Harris et al., 2009).

### Materials

Completed semi-structured questionnaires included multiple items related to symptomatic experience of COVID-19, daily functional challenges following COVID-19 diagnosis, mental health and socio-economic factors. The questionnaire included an open-ended item for respondents to report qualitatively about their experience of COVID-19 infection.

### Long COVID-19

Long COVID was defined as reporting any current symptom that developed within 3 months of a confirmed positive COVID-19 test that could not be explained by another diagnosis.

### Daily functioning

Challenges in daily functioning following first COVID-19 diagnosis was recorded through an expanded version of the Sheehan Disability Scale (Leon et al., 1997). The original SDS is a validated measure of disability experienced in three life domains – work, family and social functioning. To these three domains, we added the domains of self-care and leisure. Each item measured the respondent’s sense of impairment in the respective domain after first testing positive for COVID-19. Response options range from ‘0 / no change’ following diagnosis to ‘10 / worst possible impediment’ using a visual analog scale. The SDS has been utilized and demonstrated predictive validity and reliability in multiple settings, with varying patient populations and psychiatric outcomes (Greene et al., 2016; Kessler & Wang, 2008; Laird et al., 2017). Factor analysis revealed a strong single factor for all 5 items, accounting for 95% of underlying variance. The internal reliability of the 5-item measure was excellent (*α* = 0.91).

### Common mental disorder

Due to the range and number of measures included in the survey, we utilized the ultra-brief PHQ-4 to screen for anxiety and depression (Kroenke et al., 2009). The PHQ-4 combines previously validated 2-item measures of depression and anxiety (PHQ-2 & GAD-2), and has been established validity and reliability to predict severity of co-occurring anxiety and depression. The PHQ-4 is associated with functional impairment, disability days, healthcare use, and other psychometric measures – such as self-esteem, life satisfaction and resilience (Kroenke et al., 2010; Löwe et al., 2010). The four items demonstrated excellent reliability in the current study (*α* = 0.91) as it has in previous American, German, and Colombian populations (Kocalevent et al., 2014; Löwe et al., 2010). Response options range from 0–3 for the four items, leaving a total possible range of 0–6 for depression (PHQ-2) and 0–6 for anxiety (GAD-2). In bivariate analyses, previous thresholds validated in American and German populations for depression and anxiety were used to indicate the probable presence of depression or anxiety (scores ≥3). In bivariate analyses, depression and anxiety were treated separately, as binary indicators. In multivariable analyses, the continuous sum of responses to the PHQ-4 was used.

### Post-Traumatic Stress Disorder

Post-traumatic stress disorder (PTSD) was measured using the 4-item PC-PTSD (Cameron & Gusman, 2003). The PC-PTSD was developed with a military veteran population, but has shown predictive validity among civilian populations (Van Dam et al., 2010). The PC-PTSD screen measures the presence of intrusive thoughts and feelings during the past month provoking numbness, hypervigilance, or disruption during the previous month and related to a life experience. The 4-items utilize a binary response option for 4 symptoms of PTSD, with excellent internal reliability (KR20 = 0.84).

### Suicide ideation

Suicide ideation was measured using the 4 screening items of the Modified Scale for Suicide Ideation (Miller et al., 1986). These 4 items measure the intensity of the desire to die, will to live, active planning for ending one’s life, and passive interest in avoiding behaviors to support one’s life over the past 48 h. These 4 items are measured on a response format ranging from 0 to 3, and had a strong single factor solution with excellent internal consistency (*α* = 0.82). The intention of this study was to identify need for mental health follow-up and intervention at the population level attributable to Long COVID, rather than to conduct a lifetime prevalence study of suicide ideation and risk. As such, recent suicide ideation rather than lifelong suicide ideation was selected. The MSSI-screen adds wider variation in response options and domains assessed compared to other brief screening approaches that also demonstrate predictive validity – like the single-item approach included in depression inventories (e.g. BDI-II, item 9; Green et al., 2015) or modules of larger standardized interviews (e.g. Sheehan et al., 1998). In bivariate analyses, screening positive for suicide ideation with the need for follow-up was determined utilizing the guidance provided by Miller et al. (1986). In multivariable analyses, the screen for suicide ideation was treated as a continuous variable.

### Alcohol use disorder

Alcohol use was measured using the AUDIT-C (Bradley et al., 2007). The AUDIT-C is a 3-item measure of the frequency and quantity of alcohol consumption. The three items measured on a range from 0 to 4 (*α* = 0.74), with a validated cut-point to classify problematic drinking for men (≥4) and women (≥3). The continuous measure is used in multivariable analyses, while the problematic drinking cut-point is used in bivariate analyses.

### Satisfaction with life

Satisfaction with life was measured using the SWLS (Diener et al., 1985). The SWLS uses 5 items to assess global life satisfaction, with consistently favorable reliability and validity across multiple cultures and languages (Pavot & Diener, 2009). The SWLS is sensitive to life changes, and discriminates from other measures of emotional well-being. Life satisfaction reflects, in general, the degree of concordance between one’s expectations in life and current life appraisal. The SWLS was developed to respond to other life domains based on respondent’s life experience. Items included statements like ‘I am satisfied with my life.’ In this study, SWLS responses were recorded on a 7-point Likert-type format with higher scores reflecting higher life satisfaction and demonstrated excellent internal reliability (*α* = 0.9).

### Sociodemographic variables

Recorded sociodemographic characteristics included race/ethnicity, age, gender, and formal educational attainment. Race was recorded utilizing US Census categories – White, Black or African American, American Indian or Alaska Native, Native Hawaiian or Other Pacific Islander; similarly, ethnicity was recorded as Hispanic or not-Hispanic. Age was recorded in years of age completed at last birthday. Gender was recorded as male / female / and gender non-binary. Educational attainment reflects highest level of formal education by category.

### Economic variables

Economic variables included categorized household income, expense-to-income ratio and food insecurity. Household expense-to-income ratio was measured using an item from the National Finance Capability Survey expanded from binary to 6-point response options: ‘does your household income cover your expenses: very poorly, poorly, fairly poorly, fairly easily, easily or very easily’ (Angrisani et al., 2016). Food insecurity was measured using the FI-2, a validated two-item measure to screen for food insecurity, and included as a continuous measure (Castell et al., 2015).

The Centers for Disease Control and Prevention produces a Social Vulnerability Index (SVI) compiling 15 indicators related to economic, demographic, housing, and transportation factors. The SVI composite measure, positively associated with lower COVID-19 vaccine and higher COVID-19 mortality, is available at census block group and county levels. We paired respondent addresses to their census block group SVI, and included this as a potential covariate in all multivariable analyses.

### Health system data

Health system data included: date of first reported positive COVID-19 test to Galveston County Health District, health system-reported hospitalization, patient-reported insurance status, relationship to a primary care provider (PCP), and current vaccine status. Date of first COVID-19 test followed a clear 4-wave pattern, and was categorized accordingly. The relationship to a PCP was recorded using five options: (1) does not have PCP, (2) has PCP, but has not discussed health condition post-COVID, (3) has PCP, but is not satisfied with clinical care following COVID diagnosis, (4) has PCP, and is satisfied with care following COVID diagnosis, and (5) other. Primary insurance provider was recorded as: (1) uninsured, (2) Medicaid / Medicare, (3) Affordable Care, (4) employer-provided, and (5) other. Current vaccine status was recorded from subject self-report, and coded in the present study as never vaccinated vs. at least one vaccine dose.

### Open response

Respondents were prompted to provide an open response to the statement ‘What else would you like people to know about your experience of COVID-19, including, possibly, your experience of Long COVID symptoms?’ A total of 265 out of 655 respondents responded to this final item.

### Statistical analysis

Univariate analyses were used to overview all model variables.

Bivariate analyses compared all model variables to the reported presence of any Long COVID symptom. Kruskall-Wallis tests were used for continuous outcome variables, and Χ^2^ tests were used for binary outcome variables.

Structural equation models were used to analyze pathways between continuous variables in multivariable analyses. We were interested in the extent to which any current symptom predicts, in sequence, daily functional impairment, common mental disorder, PTSD, suicide ideation, problematic alcohol use and life satisfaction. While daily functional impairment, common mental disorder, PTSD, suicide ideation and problematic alcohol use are comorbid conditions, theoretical and prior empirical data support our hypothesized sequence. In brief, we hypothesized that (1) the presence of a health condition subsequent to SARS-CoV-2 viral infection would disrupt daily life, (2) life disruptions would be reflected in worse common mental disorders, (3) mental disorders would antagonize traumatic stress, (4) disruptions to daily life, higher common mental disorders and traumatic stress would provoke thoughts of suicide and overuse of alcohol, and (5) these mental and behavioral challenges would increase the delta between life expectation and assessment – lowering life satisfaction. Life satisfaction and suicide ideation were permitted to correlate in model specification.

Model development began with a saturated model reflecting hypothesized pathways, and used a backward stepwise model building process to produce a parsimonious model where all included variables were significant at *p* < 0.25. Control variables included all economic, sociodemographic, and health system data were assessed in each pathway. Model fit statistics were used to optimize final model performance.

### Qualitative analysis

The quantitative findings provided the basis for the deductive codes that were used to identify and extract relevant qualitative data.

### Ethical considerations

All respondents provided informed consent before participating in the public health surveillance study. Respondents received $40 for their participation. Analysis and dissemination of deidentified surveillance data was granted exempt status by the IRB at the University of Texas Medical Branch (IRB # 22-0119).

## Results

Table 1 presents univariate descriptions of model variables and considered sociodemographic and economic variables.

**Table 1.** Univariate description of model variables.

COVID-19 and primary outcome variables					Long COVID		
	*n*	Mean or %	95% CI		No	Yes	*p*-value
					Mean or %	Mean or %	
**Any current symptom (Long COVID)**	621	20.3%	0.17	0.23			
**Daily functional impairment**	618	2.51	2.3	2.72	2.1	3.79	<0.001
**Mental health outcomes**							
**Depression (%)**	657	19.3%	16.0%	22.0%	14.97%	28.33%	0.001
**Anxiety (%)**	655	26.9%	23.0%	30.0%	19.17%	47.06%	<0.001
**Common mental disorder (range: 1–4)**	657	1.76	1.69	1.82	1.61	2.11	<0.001
**Suicide ideation screen, positive**	649	14.6%	12.0%	17.0%	11.43%	21.19%	0.006
**Suicide ideation screen, (0– 8 range)**	649	60.0%	49.0%	72.0%	0.43	0.89	<0.001
**Post-traumatic stress disorder, present**	653	23.0%	20.0%	26.0%	19%	38.70%	<0.001
**Post-traumatic stress disorder, average**	620	1.14	1.02	1.25	0.91	1.69	<0.001
**Alcohol, problematic use**	619	29.7%	26.0%	33.0%			ns
**Alcohol, summative**	618	2.17	2.01	2.32			ns
**Satisfaction with Life**	652	4.87	4.76	4.98	5.01	4.5	0.001
**First reported COVID positive test**							
**Wave 1 (30 April 2020–27 October 2020)**	661	18.3%	16.0%	21.0%	16.2%	24.4%	0.02
**Wave 2 (30 October 2020–12 May 2021)**	661	38.9%	35.0%	43.0%	39.6%	39.5%	
**Wave 3 (7 June 2021–13 October 2021)**	661	23.9%	21.0%	27.0%	23.6%	25.2%	
**Wave 4 (20 October 2021–1 February 2022)**	661	18.9%	16.0%	22.0%	20.6%	10.9%	
**Any COVID-related hospitalization**	661	4.7%	3.0%	6.0%	3.2%	9.2%	0.005
**Relationship to PCP**							
**No PCP**	608	30.3%	27.0%	34.0%			ns
**PCP, but have not discussed COVID**	608	22.5%	19.0%	26.0%			
**PCP, but not satisfied re: COVID**	608	5.9%	4.0%	8.0%			
**PCP, satisfied re: COVID**	608	39.6%	36.0%	44.0%			
**Other**	608	2.3%	1.0%	3.0%			
Demographic and Socioeconomic variables							
%	*n*	mean or %	95% CI				
Race							
White	613	79.0%	76.0%	82.0%	81.3%	85.0%	ns
Black / AA	613	13.1%	10.0%	16.0%	11.5%	9.7%	
AI / AN	613	3.6%	2.0%	5.0%	5.3%	2.7%	
Asian	613	5.2%	3.0%	7.0%	1.7%	2.7%	
NH/OPI	613	0.7%	0.0%	1.0%	0.2%	0.0%	
Ethnicity							
Non-Hispanic	613	64.9%	61.0%	69.0%	66.5%	64.9%	ns
Hispanic	613	35.1%	31.0%	39.0%	33.5%	35.1%	
Gender							
Male	613	23.5%	20.0%	27.0%	26.1%	16.5%	0.01
Female	613	75.5%	72.0%	79.0%	73.0%	82.6%	
Gender non-binary	613	1.0%	0.0%	2.0%	0.1%	0.1%	
Age (yrs)	616	34.88	34.17	35.59	34.5	36.9	<0.01
Formal education completed							
Less than high school diploma	620	3.7%	3.0%	6.0%			ns
High school diploma or GED	620	17.7%	15.0%	21.0%			
Some college, but no degree	620	25.2%	22.0%	29.0%			
Associate's degree	620	14.7%	12.0%	18.0%			
Bachelor's degree	620	25.5%	22.0%	29.0%			
Master's degree	620	10.3%	8.0%	13.0%			
Professional degree	620	1.8%	1.0%	3.0%			
Doctorate	620	1.1%	1.0%	2.0%			
Income							
Less than $20,000	600	14.7%	12.0%	18.0%			ns
$20,000–$34,999	600	13.7%	11.0%	17.0%			
$35,000–$49,999	600	11.8%	10.0%	15.0%			
$50,000–$74,999	600	14.8%	12.0%	18.0%			
$75,000–$99,999	600	11.3%	9.0%	14.0%			
$100,000 or above	600	33.7%	30.0%	37.0%			
Social Vulnerability Index	658	653.0%	266.0%	1114.0%			ns
Food insecurity	613	132.0%	128.0%	137.0%	1.26	1.57	<0.001
No Insurance provider	608	22.0%	19.0%	25.0%			ns
Cover household expenses ‘poorly/very poorly’	616	12.0%	9.6%	14.9%	10.4%	19.8%	<0.01
Never vaccinated	659	40.7%	37.0%	44.0%			**ns**

Notes: Mean or percentage and 95% confidence intervals for all model variables. Right column set compares values by the absence or presence of Long COVID – any reported symptom. Statistical tests include *χ*^2^ for all categorical and binary variables, Kruskal-Wallis tests of independence for all ordinal or continuous variables. *P*-values and corresponding values only shown where *p* < 0.05.

One respondent who reported Long COVID noted that ‘I've tested positive twice, I've never regained my full smell or taste and I still don't feel as though my lungs are back in great condition. Covid has definitely changed my life and my family's life forever! (respondent #372)’

Over 20% of respondents reported they were currently experiencing a symptom attributable to COVID-19 (Long COVID). Nearly 20% of respondents reported depression, and over 1 in 4 reported anxiety. Nearly 15% of respondents (14.6%) screened positive for suicide ideation. Over 20% screened positive for PTSD, and nearly 30% reported problematic alcohol use. One respondent who reported Long COVID and a common mental disorder said that ‘It was a horrible virus and I'm glad I got better from the severe symptoms. But I still have brain fog and I don't know if I will ever be the same again’ (respondent #60).

In the open-ended response field, a participant who reported suicide ideation, a common mental disorder, and multiple other issues as part of their post-COVID experience noted that:

*Pre covid I worked 40 h work weeks as a pastry chef on my feet. Post covid I can barely stand longer than 10 min and I can't exercise at all … I now am disabled permanently (which was a hard pill to swallow [by the way]) and am relient [sic] on a cane on short journeys and wheelchair on long ones. I am also still waiting to receive disability payments through the [SSA] so while I've been waiting. I am below the poverty line and miserable because of my illness and financial instability. This sucks. This sucks so so much.* (respondent #371)

Over 40% of respondents reported they have never been vaccinated, and nearly 5% of respondents were hospitalized due to COVID-19. Over 30% of respondents did not have a primary care provider, and over 20% reported no insurance provider. In the words of one respondent, ‘[t]he Urgent care facility's [*sic*] need to inform more people how to take care of themselves and not to just quarantine. As that is how I ended up in the hospital for 8 days because I didn't know how to take care of myself.’ (respondent #754)

More than 25% of respondents reported earned less than $35,000 annual household income, and over 10% reported they could ‘poorly,’ or ‘very poorly’ cover their household expenses. More than 60% of respondents had completed an associate’s degree or less of formal education, and more than 75% of respondents were women. Thirty-five percent of respondents reported Hispanic ethnicity, and over 75% of respondents reported white race.

Daily functioning, depression, anxiety, suicidal ideation, and life satisfaction were significantly worse among respondents who reported Long COVID. Problematic alcohol use was not worse among respondents reporting Long COVID. Long COVID accounted for an additional 13% increase in depression, 28% increase in anxiety, and 10% increase in suicidal ideation.

According to one participant who reported suicide ideation and other negative factors after their experience with COVID-19, ‘For being fairly young and healthy, that was the most ill I have ever been in my life. I was sick for 10 consecutive days and has [*sic*] lasting effects for months after I tested negative. I still battle random brain fog/confusion,’ and that ‘recently I was driving to my gym and completely forgot where I was, had to pull over on I-45 to try to remember what I was doing and where I was going’ (respondent #250). For this participant and others, Long COVID reportedly affects their overall quality of life in concrete ways that they articulated in their structured survey responses and the open-ended qualitative field at the end of the survey.

**Figure 1.**
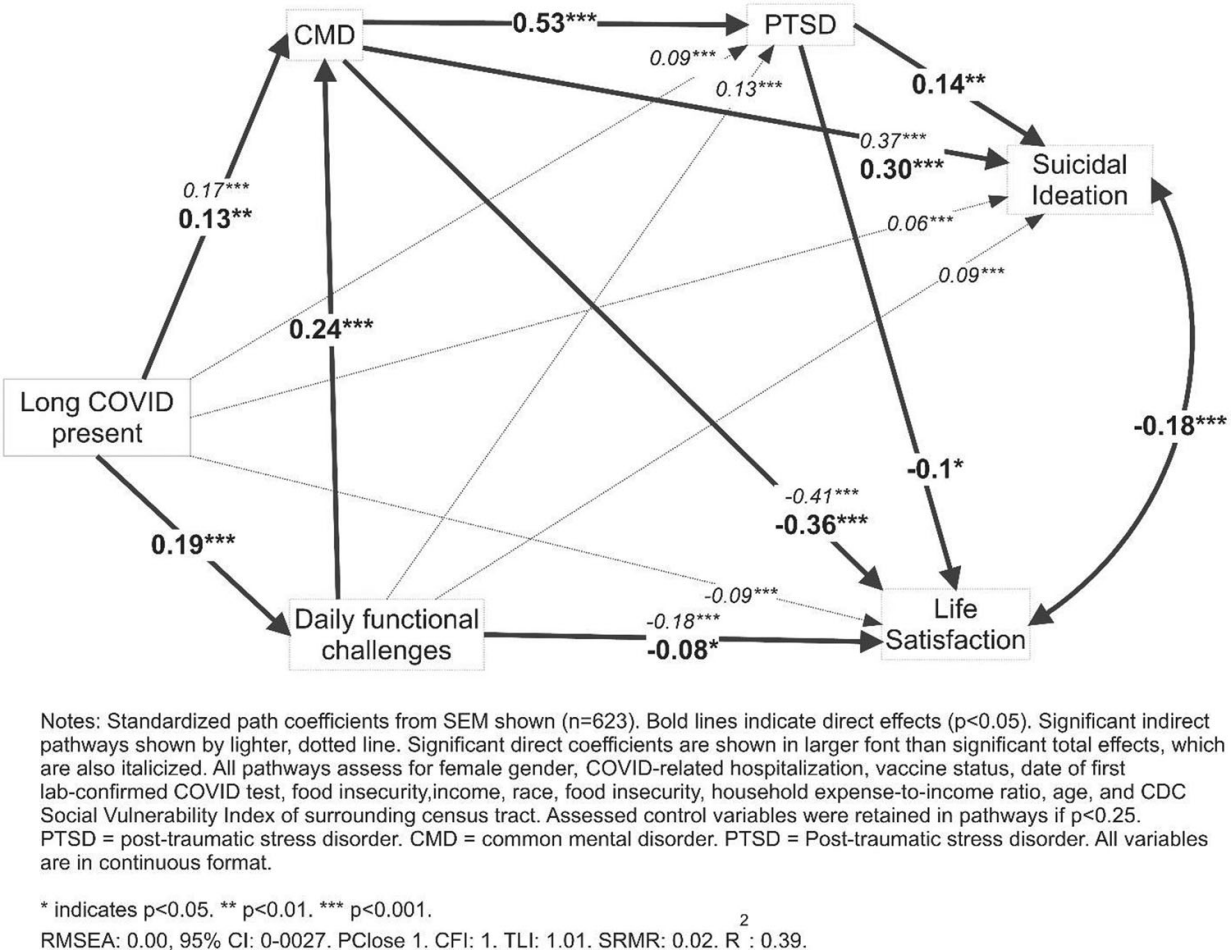
Structural Equation Model of mental health correlates and Long COVID among a community sample of adults aged 20–50 years.

Figure 1 presents the SEM-based path analysis of Long COVID and mental health outcomes, assessing and controlling for sociodemographic, economic and health system variables where associated at *p* < 0.25. Alcohol was not included in the model as it was not significantly predicted by other model variables, including Long COVID. Long COVID predicted, directly, worse daily functioning and common mental disorders. Worse daily functioning predicted, directly, lower life satisfaction and common mental disorders.

In the words of one participant who reported worse daily functioning and Long COVID:

It was a hard time to watch my family go through it alongside me, and put into perspective the severity of the pandemic. I am young and generally healthy, but had to be hospitalized because of the sickness and could barely move my body. It was found that I had a blood clot during my time at the hospital from the sickness so dealing with that has been something that affected my daily life post-covid (respondent #254).

Higher common mental disorders were significantly correlated with higher post-traumatic stress disorder and suicide ideation, and lower life satisfaction. Long COVID was indirectly correlated with higher PTSD and suicidal ideation, and lower life satisfaction.

## Discussion

The global picture of Long COVID continues emerging with greater clarity and concern. Known mental and behavioral sequelae to other chronic conditions, like chronic pain and chronic fatigue, should animate investigation into potential mental and behavioral sequelae to Long COVID. Here, we investigate correlations between Long COVID and mental health outcomes associated with other chronic conditions, revealing a disturbing picture. Significant, positive correlations persisted between Long COVID and mental health challenges after controlling for multiple demographic, socioeconomic and health system covariates. If patterns within the broader US population are consistent with those identified within study data, there may be 2.6 million adults living with depression, 5.4 million adults living with anxiety, and 1.9 million adults living with suicidal ideation attributable to Long COVID.^3^ These projections are likely to be conservative insofar as Long COVID impacts household economic conditions, which has been observed among other chronic conditions but are controlled for in the present study.

Throughout the COVID-19 pandemic, researchers have projected the end of the pandemic with varying degrees of intellectual seriousness. Ranging from epidemiological modeling by experts working carefully within their expertise in peer-reviewed journals (Jewell et al., 2020) to less careful analysis (Makary, 2021), researchers have sought to project the end of the COVID-19 pandemic. Present findings suggest immediate concerns of COVID-19 infection may be replaced by secondary, population-wide mental health effects of living with Long COVID.

We expected Long COVID to predict more alcohol use, and anticipated this correlation to be mediated by the presence of mental health challenges. This was not observed for reasons we are unable to explain. Coping strategies among people with Long COVID require further investigation, and should clarify whether these coping strategies include self-medicating by alcohol or other substance use (Leeies et al., 2010).

There is urgent need for interdisciplinary research with people with Long COVID, investigating physical and mental etiologies, coping strategies, and longitudinal impacts at individual and population levels. Interventional research should explore utilizing existing evidence-based, mHealth-supported mental health promotion strategies. Funders should also ensure ample funding for qualitative Long COVID research, as existing quantitative measures alone will not capture the diverse lived experience.

### Limitations

Cross-sectional data do not permit causal inference, and this study utilizes a hybrid of cross-sectional survey data and health system retrospective data. Research is required to assess the extent to which prior mental health challenges are reflected in the emergence of Long COVID. Such research should be multidisciplinary, including physiological, clinical and self-reported measures.

Social desirability bias likely led people to under-report potentially embarrassing outcomes like the presence of suicide ideation, depression, and anxiety, which would lead conclusions towards the null hypothesis of no association. Responding to a self-administered questionnaire in an anonymous environment, that is – not directly to a person in a face-to-face interview – likely limited the risk of social desirability bias.

Response and sampling biases are potential sources of misclassification, though how these influenced results is difficult to ascertain. Given relatively high levels of literacy and smartphone ownership among younger American adults (O’Dea, 2022), we believe it possible that respondents who were unmotivated by the incentive ($40) were as unlikely to respond as people who did not own a smartphone and could not access the survey. Respondents appeared motivated by the incentive, as demonstrated by follow-up contact prior to the funding disbursement, and may have over-represented lower income individuals. Income categories assessed in this study were not significantly associated with self-reported Long COVID symptoms, suggesting income along was inadequate to bias point estimates of Long COVID. While mental health correlates were associated with mental health correlates, dubious associations created by response or sampling bias related to income would require mental correlates also be associated with income.

## Conclusions

Study findings mirrored CDC estimates that around 20% of people who contracted COVID-19 prior to vaccination live with at least one current Long COVID symptom. This study provides nuance to this finding by considering mental health implications of living with Long COVID. As predicted from other chronic conditions, people with Long COVID report worse mental health outcomes – including higher depression, anxiety, PTSD, suicide ideation and lower life satisfaction. Urgent research is needed to understand the lived experience of people with Long COVID, and interventions that may be rapidly deployed to meet the new population-level impact of Long COVID. Further research is required to examine directionality between Long COVID development and mental health correlates.

## Data Availability

Data are available via open access: https://osf.io/d6mfz/?view_only=88a2ed2eee334e8d9124ba56c3c32d02

## Notes

1 The phenomenon of new symptoms experienced following infection with SARS-CoV-2 has received many different names: post-acute sequela of SARS-CoV-2 (PASC), long-haul COVID, Long COVID, etc. In recognition of early patient efforts to raise awareness about on-going symptomatic challenges and chosen nomenclature, this paper uses ‘Long COVID.’

2 A separate study investigates parenting and intimate partner dynamics.

3 These calculations combine CDC data that 7.5% of Americans are living with Long COVID, estimated 258.3 million adults in the United States, and higher observed rates of depression, anxiety and suicidal ideation among people with Long COVID of 13.4%, 27.9%, and 9.8% within study data.

## References

[CIT0001] Aiyegbusi, O. L., Hughes, S. E., Turner, G., Rivera, S. C., McMullan, C., & Chandan, J. S., & TLC Study Group. (2021). Symptoms, complications and management of Long COVID: a review. *Journal of the Royal Society of Medicine*, *114*(9), 428–442. 10.1177/01410768211032850 34265229 PMC8450986

[CIT0002] Angrisani, M., Kapteyn, A., & Lusardi, A. (2016). The national financial capability study: empirical findings from the American Life Panel Survey. *FINRA report*, *43*.

[CIT0003] Bossley, C. J., Kavaliunaite, E., Harman, K., Cook, J., Ruiz, G., & Gupta, A. (2022). Post-acute COVID-19 outcomes in children requiring hospitalisation. *Scientific Reports*, *121*, 1–4. 10.1038/s41598-022-12415-x PMC9113067 35581348

[CIT0004] Bradley, K. A., DeBenedetti, A. F., Volk, R. J., Williams, E. C., Frank, D., & Kivlahan, D. R. (2007). AUDIT-C as a brief screen for alcohol misuse in primary care. *Alcoholism: Clinical and Experimental Research*, *31*(7), 1208–1217. 10.1111/j.1530-0277.2007.00403.x 17451397

[CIT0005] Braun, V., & Clarke, V. (2006). Using thematic analysis in psychology. *Qualitative Research in Psychology*, *3*(2), 77–101. 10.1191/1478088706qp063oa

[CIT0006] Callard, F., & Perego, E. (2021). How and why patients made Long COVID. *Social Science & Medicine*, *268*, Article 113426. 10.1016/j.socscimed.2020.113426 PMC7539940 33199035

[CIT0007] Cameron, R. P., & Gusman, D. (2003). The primary care PTSD screen PC-PTSD: development and operating characteristics. *Primary Care Psychiatry*, *91*, 9–14.

[CIT0008] Castell, G. S., Rodrigo, C. P., de la Cruz, J. N., & Bartrina, J. A. (2015). Household food insecurity access scale HFIAS. *Nutricion Hospitalaria*, *313*, 272–278. 10.3305/nh.2015.31.sup3.8775 25719795

[CIT0009] Centanni, S. W., Bedse, G., Patel, S., & Winder, D. G. (2019). Driving the downward spiral: Alcohol-induced dysregulation of extended amygdala circuits and negative affect. *Alcoholism: Clinical and Experimental Research*, *43*(10), 2000–2013. 10.1111/acer.14178 31403699 PMC6779502

[CIT0010] Dean, B., Aguilar, D., Shapiro, C., Orr, W. C., Isserman, J. A., Calimlim, B., & Rippon, G. A. (2010). Impaired health status, daily functioning, and work productivity in adults with excessive sleepiness. *Journal of Occupational & Environmental Medicine*, 144–149. 10.1097/JOM.0b013e3181c99505 20134351

[CIT0011] Diener, E. D., Emmons, R. A., Larsen, R. J., & Griffin, S. (1985). The satisfaction with life scale. *Journal of Personality Assessment*, *49*(1), 71–75. 10.1207/s15327752jpa4901_13 16367493

[CIT0012] Epstein, S. (1995). The construction of lay expertise: AIDS activism and the forging of credibility in the reform of clinical trials. *Science, Technology, & Human Values*, *20*(4), 408–437. 10.1177/016224399502000402 11653331

[CIT0013] Fox, R. J., Crask, M. R., & Kim, J. (1988). Mail survey response rate: A meta-analysis of selected techniques for inducing response. *Public Opinion Quarterly*, *52*(4), 467–491. 10.1086/269125

[CIT0014] Garland, E. L., Froeliger, B., Zeidan, F., Partin, K., & Howard, M. O. (2013). The downward spiral of chronic pain, prescription opioid misuse, and addiction: cognitive, affective, and neuropsychopharmacologic pathways. *Neuroscience & Biobehavioral Reviews*, *37*(10), 2597–2607. 10.1016/j.neubiorev.2013.08.006 23988582 PMC3967721

[CIT0015] Gatchel, R. J. (2004). Comorbidity of chronic pain and mental health disorders: the biopsychosocial perspective. *American Psychologist*, *59*(8), 795. 10.1037/0003-066X.59.8.795 15554853

[CIT0016] Green, K. L., Brown, G. K., Jager-Hyman, S., Cha, J., Steer, R. A., & Beck, A. T. (2015). The predictive validity of the beck depression inventory suicide item. *The Journal of Clinical Psychiatry*, *7612*, Article 15048. 10.4088/JCP.14m09391 26717528

[CIT0017] Greene, T., Neria, Y., & Gross, R. (2016). Prevalence, detection and correlates of PTSD in the primary care setting: a systematic review. *Journal of Clinical Psychology in Medical Settings*, *23*(2), 160–180. 10.1007/s10880-016-9449-8 26868222

[CIT0018] Gullo, H. L., Fleming, J., Bennett, S., & Shum, D. H. (2019). Cognitive and physical fatigue are associated with distinct problems in daily functioning, role fulfilment, and quality of life in multiple sclerosis. *Multiple Sclerosis and Related Disorders*, *31*, 118–123. 10.1016/j.msard.2019.03.024 30981190

[CIT0019] Harris, P. A., Taylor, R., Thielke, R., Payne, J., Gonzalez, N., & Conde, J. G. (2009). Research electronic data capture (REDCap)—A metadata-driven methodology and workflow process for providing translational research informatics support. *Journal of Biomedical Informatics*, *42*(2), 377–381. 10.1016/j.jbi.2008.08.010 18929686 PMC2700030

[CIT0020] Jewell, N. P., Lewnard, J. A., & Jewell, B. L. (2020). Predictive mathematical models of the COVID-19 pandemic. *Jama*, *323*(19), 1893–1894. 10.1001/jama.2020.6585 32297897

[CIT0021] Kessler, R. C., & Wang, P. S. (2008). The descriptive epidemiology of commonly occurring mental disorders in the United States. *Annual Review of Public Health*, *29*(1), 115–129. 10.1146/annurev.publhealth.29.020907.090847 18348707

[CIT0022] Kocalevent, R. D., Finck, C., Jimenez-Leal, W., Sautier, L., & Hinz, A. (2014). Standardization of the Colombian version of the PHQ-4 in the general population. *BMC Psychiatry*, *141*, 1–8. 10.1186/1471-244X-14-205 PMC4223637 25037706

[CIT0023] Kroenke, K., Spitzer, R. L., Williams, J. B., & Löwe, B. (2009). An ultra-brief screening scale for anxiety and depression: the PHQ–4. *Psychosomatics*, *506*, 613–621. 10.1176/appi.psy.50.6.613 19996233

[CIT0024] Kroenke, K., Spitzer, R. L., Williams, J. B., & Löwe, B. (2010). The patient health questionnaire somatic, anxiety, and depressive symptom scales: a systematic review. *General Hospital Psychiatry*, *32*(4), 345–359. 10.1016/j.genhosppsych.2010.03.006 20633738

[CIT0025] Kuodi, P., Gorelik, Y., Zayyad, H., Wertheim, O., Wiegler, K. B., Jabal, K. A., … Edelstein, M. (2022). Association between BNT162b2 vaccination and reported incidence of post-COVID-19 symptoms: cross-sectional study 2020-21, Israel. *npj Vaccines*, *7*(1), 1–8. 36028498 10.1038/s41541-022-00526-5 PMC9411827

[CIT0026] Laird, K. T., Tanner-Smith, E. E., Russell, A. C., Hollon, S. D., & Walker, L. S. (2017). Comparative efficacy of psychological therapies for improving mental health and daily functioning in irritable bowel syndrome: A systematic review and meta-analysis. *Clinical Psychology Review*, *51*, 142–152. 10.1016/j.cpr.2016.11.001 27870997

[CIT0027] Leeies, M., Pagura, J., Sareen, J., & Bolton, J. M. (2010). The use of alcohol and drugs to self-medicate symptoms of posttraumatic stress disorder. *Depression and Anxiety*, *27*(8), 731–736. 10.1002/da.20677 20186981

[CIT0028] Leon, A. C., Olfson, M., Portera, L., Farber, L., & Sheehan, D. V. (1997). Assessing psychiatric impairment in primary care with the Sheehan Disability Scale. *The International Journal of Psychiatry in Medicine*, *27*(2), 93–105. 10.2190/T8EM-C8YH-373N-1UWD 9565717

[CIT0029] Li, Z., Zheng, C., Duan, C., Zhang, Y., Li, Q., Dou, Z., … Xia, W. (2020). Rehabilitation needs of the first cohort of post-acute COVID-19 patients in Hubei, China. *European Journal of Physical and Rehabilitation Medicine*, *563*, 339–344. 10.23736/S1973-9087.20.06298-X 32672029

[CIT0030] Löwe, B., Wahl, I., Rose, M., Spitzer, C., Glaesmer, H., Wingenfeld, K., … Brähler, E. (2010). A 4-item measure of depression and anxiety: validation and standardization of the Patient Health Questionnaire-4 PHQ-4 in the general population. *Journal of Affective Disorders*, *1221-2*, 86–95. 10.1016/j.jad.2009.06.019 19616305

[CIT0031] Makary, M. Feb. 18, 2021. We’ll have herd immunity by April. Wall Street Journal. Accessed June 28, 2022. https://www.wsj.com/articles/well-have-herd-immunity-by-april-11613669731

[CIT0032] Manning, K., Bakhshaie, J., Shepherd, J. M., Jones, J., Timpano, K. R., Viana, A. G., & Zvolensky, M. J. (2019). Fatigue severity and emotion dysregulation: roles in mental health among trauma exposed college students. *Fatigue: Biomedicine, Health & Behavior*, *7*(4), 181–195. 10.1080/21641846.2019.1661942

[CIT0033] Mehandru, S., & Merad, M. (2022). Pathological sequelae of long-haul COVID. *Nature Immunology*, *23*(2), 194–202. 10.1038/s41590-021-01104-y 35105985 PMC9127978

[CIT0034] Mendelson, M., Nel, J., Blumberg, L., Madhi, S. A., Dryden, M., Stevens, W., & Venter, F. W. D. (2021). Long-COVID: An evolving problem with an extensive impact. *SAMJ: South African Medical Journal*, *1111*, 10–12. 10.7196/SAMJ.2020.v111i11.15433 33403997

[CIT0035] Miller, I. W., Norman, W. H., Bishop, S. B., & Dow, M. G. (1986). The modified scale for suicidal ideation: Reliability and validity. *Journal of Consulting and Clinical Psychology*, *54*(5), 724. 10.1037/0022-006X.54.5.724 3771893

[CIT0036] Morasco, B. J., Lovejoy, T. I., Lu, M., Turk, D. C., Lewis, L., & Dobscha, S. K. (2013). The relationship between PTSD and chronic pain: mediating role of coping strategies and depression. *Pain*, *154*(4), 609–616. 10.1016/j.pain.2013.01.001 23398939 PMC3609886

[CIT0037] Nater, U. M., Lin, J. M. S., Maloney, E. M., Jones, J. F., Tian, H., Boneva, R. S., Raison, C. L., Reeves, W. C., Heim, C. (2009). Psychiatric comorbidity in persons with chronic fatigue syndrome identified from the Georgia population. *Psychosomatic Medicine*, *71*(5), 557–565. 10.1097/PSY.0b013e31819ea179 19414619

[CIT0038] National Center for Health Statistics. (June 22, 2022). “Nearly one in five American adults who have had COVID-19 still have ‘Long COVID.’” Centers for Disease Control and Prevention. Retrieved June 23, 2022. https://www.cdc.gov/nchs/pressroom/nchs_press_releases/2022/20220622.htm

[CIT0039] O’Dea, S. (2022). Smartphone penetration in the U.S. as a share of population 2010-2021). Statista. Retrieved December 16, 2022. https://www.statista.com/statistics/201183/forecast-of-smartphone-penetration-in-the-us/

[CIT0040] Oishi, S., Diener, E., Lucas, R. E., & Suh, E. M. (2009). Cross-cultural variations in predictors of life satisfaction: Perspectives from needs and values. In *Culture and well-being* (pp. 109–127). Springer.

[CIT0041] Okifuji, A., & Turk, D. C. (2016). Chronic pain and depression: vulnerability and resilience. In *Neuroscience of pain, stress, and emotion*. Academic Press. pp. 181–201.

[CIT0042] Outcalt, S. D., Kroenke, K., Krebs, E. E., Chumbler, N. R., Wu, J., Yu, Z., & Bair, M. J. (2015). Chronic pain and comorbid mental health conditions: independent associations of posttraumatic stress disorder and depression with pain, disability, and quality of life. *Journal of Behavioral Medicine*, *38*(3), 535–543. 10.1007/s10865-015-9628-3 25786741

[CIT0043] Pavot, W., & Diener, E. (2009). Review of the satisfaction with life scale. In *Assessing well-being* (pp. 101–117). Springer.

[CIT0044] Penetra, S. L., da Silva, M. F., Resende, P., Pina-Costa, A., Santos, H. F., Guaraldo, L., Calvet, G. A., Ogrzewalska, M., Arantes, I., Zukeram, K., de Araújo, M. F., Lima, A. B. M., Lopes, R. S., Lira-Silva, L. R., Moraes, I. V., Wakimoto, M. D., Fuller, T. L., Gabaglia, C. R., Espíndola, O. M., Bonaldo, M. C., & Brasil, P. (2022). Post-acute COVID-19 syndrome after reinfection and vaccine breakthrough by the SARS-CoV-2 Gamma variant in Brazil. *International Journal of Infectious Diseases*, *114*, 58–61. 10.1016/j.ijid.2021.10.048 34757006 PMC8553653

[CIT0045] Remien, R. H., Stirratt, M. J., Nguyen, N., Robbins, R. N., Pala, A. N., & Mellins, C. A. (2019). Mental health and HIV/AIDS. *AIDS*, *33*(9), 1411. 10.1097/QAD.0000000000002227 30950883 PMC6635049

[CIT0046] Say, D., Crawford, N., McNab, S., Wurzel, D., Steer, A., & Tosif, S. (2021). Post-acute COVID-19 outcomes in children with mild and asymptomatic disease. *The Lancet Child & Adolescent Health*, *5*(6), e22–e23. 10.1016/S2352-4642(21)00124-3 33891880 PMC8057863

[CIT0047] Sheehan, D. V., Lecrubier, Y., Sheehan, K. H., Amorim, P., Janavs, J., Weiller, E., … Dunbar, G. C. (1998). The Mini-International Neuropsychiatric Interview MINI: the development and validation of a structured diagnostic psychiatric interview for DSM-IV and ICD-10. *Journal of Clinical Psychiatry*, *5920*, 22–33. 9881538

[CIT0048] Shim, E. J., Lee, S. H., Kim, N. J., Kim, E. S., Bang, J. H., Sohn, B. K., Park, H. Youn, Son, K.-L., Hwang, H., Lee, K.-M., & Hahm, B. J. (2019). Suicide risk in persons with HIV/AIDS in South Korea: a partial test of the interpersonal theory of suicide. *International Journal of Behavioral Medicine*, *26*(1), 38–49. 10.1007/s12529-018-9749-5 30255219

[CIT0049] Tohamy, D., Sharaf, M., Abdelazeem, K., Saleh, M. G., Rateb, M. F., Soliman, W., Kedwany, S. M, Omar Abdelmalek, M., Medhat, M. A., Tohamy, A. M., & Mahmoud, H. (2021). Ocular manifestations of post-acute COVID-19 syndrome, upper Egypt early report. *Journal of Multidisciplinary Healthcare*, *14*, 1935. 10.2147/JMDH.S323582 34326644 PMC8315779

[CIT0050] Tough, H., Siegrist, J., & Fekete, C. (2017). Social relationships, mental health and wellbeing in physical disability: a systematic review. *BMC Public Health*, *171*, 1–18. 10.1186/s12889-017-4308-6 PMC5422915 28482878

[CIT0051] Van Dam, D., Ehring, T., Vedel, E., & Emmelkamp, P. M. (2010). Validation of the Primary Care Posttraumatic Stress Disorder screening questionnaire (PC-PTSD) in civilian substance use disorder patients. *Journal of Substance Abuse Treatment*, *39*(2), 105–113. 10.1016/j.jsat.2010.05.005 20598826

[CIT0052] Vanichkachorn, G., Newcomb, R., Cowl, C. T., Murad, M. H., Breeher, L., Miller, S., … Higgins, S. (2021, July). Post–COVID-19 syndrome long haul syndrome: description of a multidisciplinary clinic at Mayo Clinic and characteristics of the initial patient cohort. In *Mayo clinic proceedings*, Vol. 96, No. 7 (pp. 1782–1791). Elsevier. 34218857 10.1016/j.mayocp.2021.04.024 PMC8112396

[CIT0053] Wilson, K. G., Heenan, A., Kowal, J., Henderson, P. R., McWilliams, L. A., & Castillo, D. (2017). Testing the interpersonal theory of suicide in chronic pain. *The Clinical Journal of Pain*, *33*(8), 699–706. 10.1097/AJP.0000000000000451 27768608

[CIT0054] Wilson, K. G., Kowal, J., Henderson, P. R., McWilliams, L. A., & Péloquin, K. (2013). Chronic pain and the interpersonal theory of suicide. *Rehabilitation Psychology*, *58*(1), 111. 10.1037/a0031390 23438008 PMC3998981

